# Assessment of Aspartate and Bicarbonate Produced From Hyperpolarized [1-^13^C]Pyruvate as Markers of Renal Gluconeogenesis

**DOI:** 10.3389/fphys.2021.792769

**Published:** 2021-12-10

**Authors:** Hikari A. I. Yoshihara, Arnaud Comment, Juerg Schwitter

**Affiliations:** ^1^Laboratory for Functional and Metabolic Imaging, Institute of Physics, Swiss Federal Institute of Technology (EPFL), Lausanne, Switzerland; ^2^Cancer Research UK Cambridge Institute, University of Cambridge, Cambridge, United Kingdom; ^3^General Electric Healthcare, Chalfont St Giles, United Kingdom; ^4^Division of Cardiology, Lausanne University Hospital (CHUV), Lausanne, Switzerland; ^5^Cardiac MR Center, Lausanne University Hospital (CHUV), University of Lausanne (UNIL), Lausanne, Switzerland

**Keywords:** dynamic nuclear polarization, magnetic resonance spectroscopy – MRS, metabolic imaging, 3-mercaptopicolinic acid, pyruvate dehydrogenase – PDH, renal metabolism, phosphoenolpyruvate carboxykinase – PEPCK

## Abstract

As both a consumer and producer of glucose, the kidney plays a significant role in glucose homeostasis. Measuring renal gluconeogenesis requires invasive techniques, and less invasive methods would allow renal gluconeogenesis to be measured more routinely. Magnetic resonance spectroscopy and imaging of infused substrates bearing hyperpolarized carbon-13 spin labels allows metabolism to be detected within the body with excellent sensitivity. Conversion of hyperpolarized 1-^13^C pyruvate in the fasted rat liver is associated with gluconeogenic flux through phosphoenolpyruvate carboxykinase (PEPCK) rather than pyruvate dehydrogenase (PDH), and this study tested whether this was also the case in the kidney. The left kidney was scanned in fed and overnight-fasted rats either with or without prior treatment by the PEPCK inhibitor 3-mercaptopicolinic acid (3-MPA) following infusion of hyperpolarized 1-^13^C pyruvate. The ^13^C-bicarbonate signal normalized to the total metabolite signal was 3.2-fold lower in fasted rats (*p* = 0.00073) and was not significantly affected by 3-MPA treatment in either nutritional state. By contrast, the normalized [1-^13^C]aspartate signal was on average 2.2-fold higher in the fasted state (*p* = 0.038), and following 3-MPA treatment it was 2.8-fold lower in fed rats and 15-fold lower in fasted rats (*p* = 0.001). These results confirm that, unlike in the liver, most of the pyruvate-to-bicarbonate conversion in the fasted kidney results from PDH flux. The higher conversion to aspartate in fasted kidney and the marked drop following PEPCK inhibition demonstrate the potential of this metabolite as a marker of renal gluconeogenesis.

## Introduction

**FIGURE 1:**
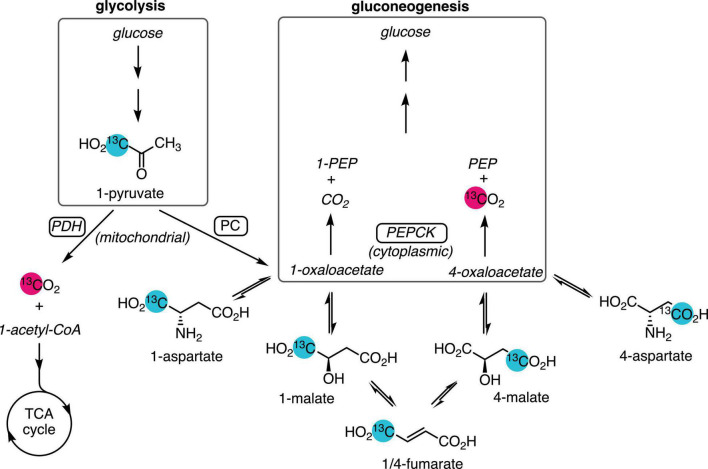
Metabolic pathways yielding ^13^C-labeled bicarbonate from hyperpolarized [1-^13^C]pyruvate. Pyruvate produced from glycolysis or lactate is oxidized by PDH and enters the TCA cycle, yielding [^13^C]CO_2_, which is reversibly converted to [^13^C]bicarbonate. Anaplerotic flux by PC in the mitochondrion converts [1-^13^C]pyruvate to [1-^13^C]oxaloacetate, which exchanges with the more abundant aspartate and malate pools. Dehydration of malate to fumarate and rehydration results in labeling the 4-carbon. Export to the cytosol *via* the malate–aspartate shuttle and conversion of [4-^13^C]oxaloacetate by gluconeogenic flux through PEPCK yields PEP and [^13^C]CO_2_. Unobserved metabolites are rendered in italic text. Site of ^13^C-labeling is indicated by the numeral preceding the metabolite name.

Along with the liver, the kidney synthesizes glucose and plays an important role in glucose homeostasis. Renal gluconeogenesis makes a greater contribution during starvation conditions, where it is estimated to be responsible for as much as 40–45% of total glucose production (Soty et al., 2017), and the diabetic kidney also has augmented gluconeogenic flux (Alsahli and Gerich, 2017). Renal gluconeogenesis is usually measured by the arterial–venous difference in glucose concentration and isotope enrichment across the kidney, which requires invasive sampling from the renal vein (Meyer et al., 1998; Stumvoll et al., 1998). Less invasive methods to estimate renal gluconeogenesis would allow this parameter to be measured more routinely in medicine and research. The large but transient signal enhancement afforded by carbon-13 spin hyperpolarization enables the sensitive, rapid detection of the metabolism of infused labeled substrates using magnetic resonance spectroscopy and imaging. In most tissues, the conversion of hyperpolarized [1-^13^C]pyruvate to [^13^C]bicarbonate is attributed to flux through the pyruvate dehydrogenase (PDH) complex (Comment and Merritt, 2014), proceeding to the tricarboxylic acid (TCA) cycle and ATP production *via* oxidative phosphorylation. In the perfused mouse liver, labeled bicarbonate is also produced by gluconeogenic flux through phosphoenolpyruvate carboxykinase (PEPCK) (Merritt et al., 2011; Moreno et al., 2015), and it is the main source of this signal in the fasted rat liver (Can et al., 2021). Bicarbonate is also a metabolic product of hyperpolarized pyruvate in the kidney (Kohler et al., 2007; Laustsen et al., 2013; Baligand et al., 2017), as is aspartate (von Morze et al., 2017), which results from the reversible transamination of oxaloacetate produced by pyruvate carboxylase (PC) (Figure 1). As oxaloacetate is a substrate of PEPCK and a key intermediate in gluconeogenesis, the aspartate signal is a potential marker of gluconeogenic flux. This study was undertaken to determine whether, like in the fasted liver, the conversion of hyperpolarized pyruvate to bicarbonate was due to PEPCK flux and also to assess the suitability of pyruvate-to-aspartate conversion as a marker of gluconeogenic flux.

## Materials and Methods

### Animal Experiments

All animal experiments were conducted in accordance with federal regulations (Swiss Federal Act on Animal Protection) and were authorized by the Service de la consommation et des affaires vétérinaires (SCAV – EXPANIM) of the Canton of Vaud, Switzerland. Male Wistar (*n* = 14, 250.9 ± 15.9 g) were anesthetized with isoflurane in a 1:1 mixture of air and oxygen and fitted with catheters in the femoral arteries and vein. Blood was sampled from an arterial catheter following each hyperpolarized experiment and analyzed immediately using an epoc system with blood gas, electrolyte and metabolite measurement cards (Siemens Healthcare AG, Zurich, Switzerland). The respiration rate was maintained around 60 breaths per minute by adjusting the isoflurane level, and body temperature was kept between 37.5 and 38.5°C using warm water circulating through silicone tubing placed next to the rat. Breathing was monitored with a pneumatic pillow coupled to a pressure transducer and body temperature with a rectal probe. Both were connected to a monitoring system (SA Instruments, Inc., Stony Brook, NY, United States). The fasted group was deprived of food overnight for 12 h. When used, the PEPCK inhibitor 3-mercaptopicolinic acid (3-MPA) (TRC, Toronto, ON, Canada) was administered intraperitoneally (100 mg/kg, neutralized, in PBS) 1 h prior to the hyperpolarized pyruvate infusion. In five experiments, the rats were scanned twice with hyperpolarized pyruvate, with 3-MPA treatment prior to the second infusion. Animals were sacrificed after scanning under 4% isoflurane anesthesia by intravenous sodium pentobarbital.

### Pyruvate Polarization

Ten microliters of 1-^13^C pyruvic acid (Sigma-Aldrich Chemie GmbH, Buchs SG, Switzerland) formulated with 21 mM OX63 trityl radical (Albeda Research ApS, Copenhagen, Denmark) was frozen in a sample cup, along with 13 μl of 10 M NaOH for neutralization, and polarized in a custom-built instrument (7 T and 1.05 K) (Comment et al., 2007; Cheng et al., 2013) with microwave irradiation (196.59 GHz, 55 mW). The sample was dissolved with 5.5 ml of hot 47 mM sodium phosphate, 100 mM NaCl, 2.7 mM KCl, 0.3 mM EDTA, pH 7.4 in D_2_O, rapidly transferred to the scanner magnet, and 1.3 ml was infused intravenously (∼0.13 mmol/kg).

### MR Acquisition

Data were acquired in a 9.4 T horizontal-bore magnet (Magnex Scientific, Abingdon, United Kingdom) with a VNMRS console (Varian, Palo Alto, CA, United States) and VnmrJ 3.2 (Agilent Technologies, Santa Clara, CA, United States). A surface coil equipped with a single loop tuned to ^1^H (400.2 MHz) and two 16 mm loops in quadrature tuned to ^13^C (100.67 MHz) was placed over the left kidney (to avoid signals from the liver), its position verified by ^1^H GEMS MRI, and shimmed using FASTESTMAP (Gruetter and Tkác, 2000). A series of ^13^C magnetic resonance spectra were acquired starting with the pyruvate infusion (∼2.9 s repetition time, with respiratory gating and cardiac triggering, 30° BIR4 adiabatic excitation, 20161.3 Hz spectral width, 8258 complex points, WALTZ-16 ^1^H decoupling).

### Data Analysis and Statistics

Spectral signals were fit using Bayes (Washington University, St. Louis, MO, United States^1^). Both the individual spectral time series and sums of spectra containing hyperpolarized signal from each infusion were analyzed. The metabolite areas under the curve for each series were calculated using the signal amplitudes and spectrum acquisition times. Metabolite signals were analyzed normalized to both the sum of all hyperpolarized signals and to the sum of all metabolite signals. Statistical significance was assessed by two-way ANOVA in R^2^ with Bonferroni correction to account for multiple comparisons. Graphical error bars and values in the text and tables report the mean ± SD.

## Results

**FIGURE 2:**
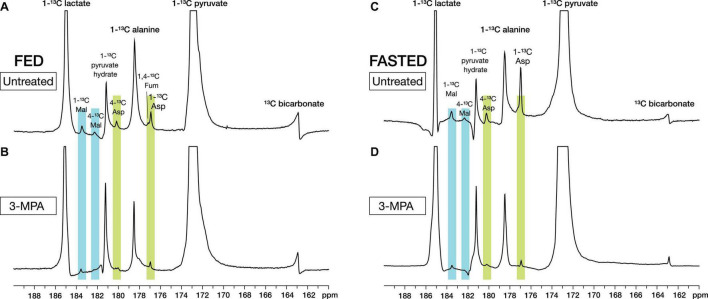
Representative high-resolution spectra of *in vivo* renal metabolism of infused hyperpolarized [1-^13^C]pyruvate in fed **(A,B)** and fasted **(C,D)** rats. The metabolites [1-^13^C]lactate, [1-^13^C]alanine, [^13^C]bicarbonate, [1-^13^C]- and [4-^13^C]aspartate, [1-^13^C]- and [4-^13^C]malate, and [1/4-^13^C]fumarate can be resolved. Treatment with the PEPCK inhibitor 3-MPA results in visibly decreased conversion to aspartate and malate. The spectra shown are sums of individual metabolite-containing spectra following a single infusion and are scaled to 9× the height of the pyruvate peak.

With metabolite spectral line widths less than 20 Hz, the smaller signals of the pyruvate metabolites aspartate and malate were readily detected along with [1-^13^C]lactate, [1-^13^C]alanine, and [^13^C]bicarbonate (Figure 2). With alanine referenced to 179.48 ppm (Wishart et al., 2018), [1-^13^C]aspartate appeared at 176.92 ppm and [1-^13^C]malate at 183.51 ppm, as well as smaller peaks corresponding to [4-^13^C]aspartate at 180.20 ppm and [4-^13^C]malate at 182.29 ppm from the reversible conversion to fumarate and resulting label scrambling (Figure 1). [1,4-^13^C]Fumarate at 177.19 ppm was also visible in some experiments. Of the smaller metabolite peaks, only the aspartate C1 signal yielded reliable fits in all experiments and was suitable for further analysis. However, the malate C1 signal and the malate and aspartate C4 peaks in the absence of 3-MPA treatment could be fit in most cases (Supplementary Figure 4).

### Effect of Fasting on Renal Metabolism of Hyperpolarized [1-^13^C]Pyruvate

**FIGURE 3:**
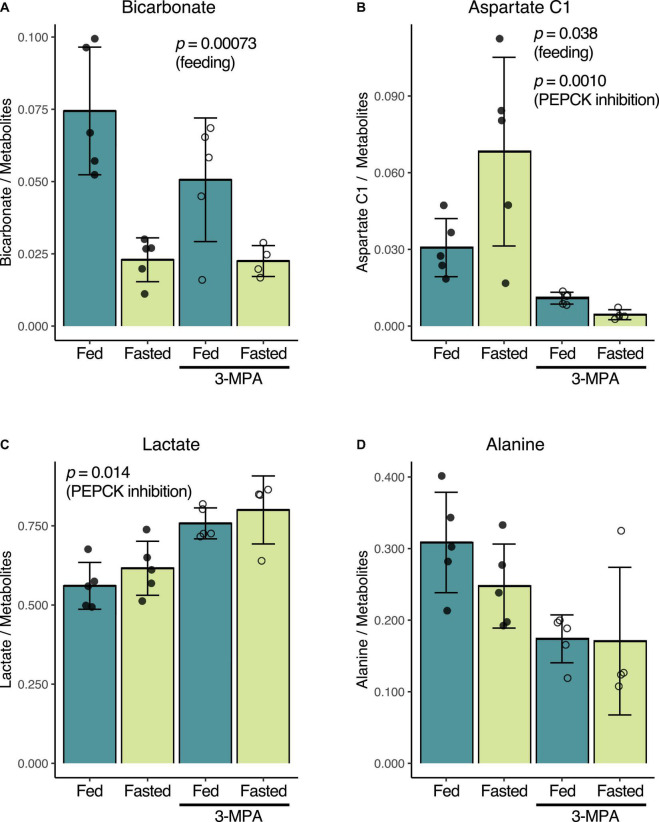
Effect of fasting and PEPCK inhibition by 3-MPA on renal conversion of [1-^13^C]pyruvate to bicarbonate **(A)**, aspartate **(B)**, lactate **(C)**, and alanine **(D)**. Metabolite-to-total metabolite signal ratios are calculated from the fitted spectral amplitudes of summed spectra from each infusion series. Filled dots represent values from untreated rats, while open circles are from 3-MPA-treated rats. The indicated *p*-values were calculated by two-way ANOVA, and they note the factor responsible for the significant difference.

Consistent with prior reports (Laustsen et al., 2014; von Morze et al., 2017), fasting and the resulting decrease in insulin levels results in lower renal conversion of [1-^13^C]pyruvate to [^13^C]bicarbonate. Normalized to the total metabolite signal of the summed spectra, the untreated fed bicarbonate signal of 0.074 ± 0.022 decreased to 0.023 ± 0.008 in the untreated fasted group (*p* = 0.00073) (Figure 3A). Conversely, [1-^13^C]aspartate signal increased from 0.031 ± 0.011 in the fed group to 0.068 ± 0.037 in the fasted rats (*p* = 0.038) (Figure 3B). The fed rats had a slightly higher fraction of alanine (Figure 3D) and lower lactate (Figure 3C), but these differences were not significant.

### Effect of 3-Mercaptopicolinic Acid on Metabolism of Hyperpolarized [1-^13^C]Pyruvate

While 3-MPA treatment did not affect the normalized [^13^C]bicarbonate signal in either the fed or fasted states (Figure 3A), it resulted in a significant decrease in the aspartate C1 signal, which was 2.8-fold lower in the fed case and 15-fold lower in fasted rats (*p* = 0.0010) (Figure 3B). Lactate C1 as a fraction of the total metabolite signal was 1.3-fold higher (*p* = 0.014) (Figure 3C) in the 3-MPA-treated rats, and alanine C1 correspondingly trended downward (Figure 3D).

Similar metabolic differences between the groups were also obtained when normalizing the summed spectra metabolite signals to the total hyperpolarized signal including pyruvate (“total carbon”) (Supplementary Figure 1) or using the dynamic spectral signal area-under-the-curve, normalized either to the total metabolite signal or total carbon (Supplementary Figures 2, 3). However, the effect of 3-MPA on the lactate signal normalized to total carbon was not significantly different even though the mean change was almost identical.

### Effect of Fasting and 3-Mercaptopicolinic Acid Treatment on Blood Glucose and Lactate

Fasting and treatment with 3-MPA had the expected effect on blood glucose and lactate levels (Table 1). Fasting glucose and lactate were lower in fasted rats, but they were not significantly different. 3-MPA treatment did not significantly change glucose and lactate levels in fed rats, but it induced hypoglycemia and a large increase in lactate in fasted rats.

**TABLE 1:** Blood glucose and lactate levels in fed and overnight fasted rats, with and without 3-MPA treatment.

Blood metabolite (mg/dl)	Fed	Fasted	Fed 3-MPA	Fasted 3-MPA	*p*-value, effect of nutrition	*p*-value, effect of 3-MPA	*p*-value, interaction
Glucose	160 ± 19	119 ± 31	147 ± 53	22 ± 3*	0.097	0.00074	0.019
Lactate	14.1 ± 9.2	10.6 ± 4.1	21.0 ± 5.5	32.1 ± 3.5	0.39	7.9 × 10^–5^	0.019

*The epoc glucose lower limit of quantitation is 20 mg/dl; and two of four readings were nominally 19.8 mg/ml. These values may therefore be less accurate.

## Discussion

These experiments provide additional evidence for the pathways responsible for the conversion of hyperpolarized [1-^13^C]pyruvate to bicarbonate in the kidney and demonstrate the potential utility of aspartate as a marker of renal gluconeogenesis. The high spectral resolution and sensitivity afforded by surface-coil-localized spectroscopy coupled with highly (>60%) polarized ^13^C pyruvate (Yoshihara et al., 2016) provide sufficient signal to reliably detect the aspartate C1 signal even after 3-MPA treatment. The fumarate and malate C1 signals and the malate and aspartate C4 peaks may reveal additional metabolic differences, but their signals would need to be further enhanced in order to be usable in all the conditions tested here.

### Hyperpolarized [^13^C]Bicarbonate and Pyruvate Dehydrogenase Flux

The substantial contribution of PEPCK flux to hyperpolarized pyruvate-to-bicarbonate conversion in the fasted rat liver raised the question whether a similar process was operating in the kidney, given its gluconeogenic function. The marked decrease of pyruvate-to-bicarbonate conversion with fasting and the lack of an effect by 3-MPA treatment shown here clearly demonstrate that any contribution by PEPCK is minor, and indicate that most, if not all, of the bicarbonate is due to PDH flux. The conversion of labeled pyruvate to bicarbonate *via* PEPCK depends on label exchange to [4-^13^C]oxaloacetate, which involves at least five steps (Figure 1), and it is more challenging to detect in the presence of PDH flux. The [4-^13^C]malate signal provides an indication of the amount of label exchange, and it is much more prominent in the liver than the kidney. Whereas the malate C4 signal normalized to the total metabolite signal was ∼0.025 in the fasted liver (Can et al., 2021) it was considerably lower in the kidney, at 0.007 or less (Supplementary Figure 4). Lower production of [4-^13^C]oxaloacetate in the kidney may therefore account for the apparent lack of [^13^C]bicarbonate resulting from PEPCK flux.

### Hyperpolarized [1-^13^C]Aspartate as a Marker of Gluconeogenesis

The increased pyruvate-to-aspartate conversion in the fasted rat kidney corresponds to the PC flux required to balance the increase in PEPCK flux in this metabolic state. This, and the greatly diminished production of labeled aspartate following inhibition of PEPCK, particularly in the fasted state, suggests that this signal provides a readout of renal gluconeogenic flux. The decreased aspartate and malate signals following 3-MPA treatment are very likely not due to a decrease in their pool size since their renal concentrations increase in fed (Bennett and Alleyne, 1976) and starved rats following treatment, with aspartate at 3.9-fold and malate at 7.1-fold their untreated levels (Vinay et al., 1980). The increased malate levels may result in the lower labeling of malate and aspartate by inhibiting PC (Scrutton and White, 1974).

The treated fed rats had higher aspartate labeling compared to the treated fasted group, so the aspartate signal may not precisely correspond to gluconeogenesis-driven PC flux, assuming complete or equivalent levels of PEPCK inhibition was achieved in both cases. PC is widely expressed throughout the rat nephron, with activity in the proximal tubule twice that in the distal tubule (Salto et al., 1991). Similarly, aspartate aminotransferase (AST) activity, which interconverts oxaloacetate and aspartate, is also widely expressed, with its activity highest in the distal straight and distal convoluted tubule and slightly lower in the proximal tubule (Chan et al., 1979). Nonetheless, the large decreases in normalized aspartate signal following 3-MPA treatment are consistent with most of the labeled aspartate resulting from gluconeogenic PC flux and therefore occurring in the proximal tubule. The malate–aspartate shuttle (Borst, 2020) plays a critical role in gluconeogenesis from pyruvate by transporting oxaloacetate equivalents from the mitochondria to the cytosol and serving as a bridge between the main sites of PC and PEPCK activity. The decreased aspartate therefore likely results from decreased aspartate turnover or shuttle activity secondary to PEPCK inhibition.

The considerable variation in the normalized aspartate signal in fasted rats (Figure 3B) may reflect its sensitivity to small metabolic differences. But the degree to which hyperpolarized pyruvate-to-aspartate conversion can serve as a general marker of renal gluconeogenesis would need to be validated in a study that measures and relates the pyruvate metabolism to independent measures of renal gluconeogenesis. Other conditions with increased renal gluconeogenesis, such as diabetes, starvation, and acidosis, may result in even greater production of labeled aspartate. Given its lower intensity and proximity to the alanine peak, resolving the aspartate spectral peak requires careful adjustment of magnetic field homogeneity, and it may be difficult to quantitate by spectroscopic imaging. It is nonetheless encouraging that it can be resolved in the rat kidney with a 3T clinical scanner (von Morze et al., 2017).

### Effects of 3-Mercaptopicolinic Acid on Other Metabolites

Although the decreased conversion of pyruvate to alanine with 3-MPA treatment did not reach the threshold for statistical significance by two-way ANOVA, there are clear differences, and the exclusion of the one high outlying point in the fasted 3-MPA group (Figure 3D) would result in a significant difference. A *t*-test comparing the untreated and treated fed groups alone yields an uncorrected *p*-value of 0.009, and one can reasonably anticipate seeing lower alanine with 3-MPA treatment in a study with higher statistical power.

### Metabolic Compartmentation in the Nephron

**FIGURE 4:**
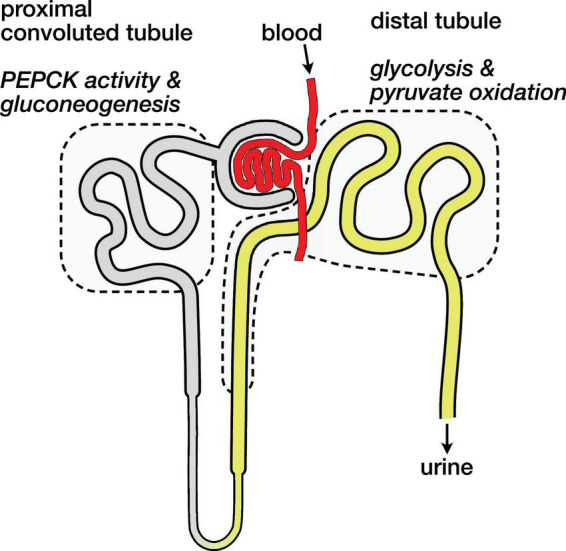
Schematic diagram of nephron showing main compartments for gluconeogenesis and pyruvate oxidation. Blood carrying hyperpolarized [1-^13^C]pyruvate is filtered and pyruvate enters cells both *via* the tubular lumen (gray and yellow) and from the surrounding capillaries (not shown). The proximal convoluted tubule is the main site of renal gluconeogenesis. The distal tubule is the main site of renal glucose and lactate oxidation, and labeled bicarbonate resulting from PDH activity is attributed to this region.

The kidney is an anatomically and metabolically complex organ, and different segments of the nephron are functionally distinct and express different ensembles of enzymes to support their metabolic roles (Schmidt and Guder, 1976; Guder and Ross, 1984). The proximal convoluted tubule is the main site of renal gluconeogenesis, while the distal tubule uses oxidizes glucose and lactate as fuel (Klein et al., 1981). These metabolic compartments are schematically illustrated in Figure 4. The observed malate and aspartate are then largely produced in the proximal convoluted tubule, and the distal tubule is likely the main site of bicarbonate production by PDH flux.

The distinct metabolic roles of these nephron segments raises some potential caveats regarding the normalization of the hyperpolarized signals. If most of the filtered pyruvate is reabsorbed in the proximal tubule (Jorgensen and Sheikh, 1988), the pyruvate metabolism attributed to the distal tubule would then result mainly from pyruvate delivered by the capillaries (Ullrich et al., 1984) rather than the filtrate in the lumen. These different routes of substrate delivery may result in different capacities and rates of pyruvate uptake in the various tubule cell populations, and they are another mechanism that may affect the apparent pyruvate metabolism *via* one pathway over another. With the mean pyruvate signal ranging from 67 to 70% of the total ^13^C signal, a gross change in pyruvate uptake is not apparent in the fasted or 3-MPA treated groups, and the simplifying assumption of analyzing the sum renal metabolites is useful.

### Limitations of Study

In addition to the important limitation imposed by the semi-quantitative nature of hyperpolarized ^13^C metabolism *in vivo*, the current study relies on the placement of the surface coil to select the renal signals. The left kidney was scanned in order to be better isolated from any signals from the liver, but the coil may also detect signals from the nearby spleen. Additionally, the non-uniform sensitivity of the surface coil results in the closer cortical regions of the kidney having a greater contribution to the measured metabolite signals.

In summary, we demonstrate that the conversion of hyperpolarized [1-^13^C]pyruvate to aspartate in the rat kidney is increased in the fasted state and greatly decreased following PEPCK inhibition, making aspartate a promising metabolic marker of renal gluconeogenesis. By contrast, the decreased renal conversion of pyruvate to bicarbonate in the fasted state was not significantly influenced by inhibition of PEPCK, unlike the case in rat liver, providing further evidence that most of hyperpolarized bicarbonate production in the kidney is due to flux through PDH.

## Data Availability Statement

The raw data supporting the conclusions of this article will be made available by the authors, without undue reservation.

## Ethics Statement

The animal study was reviewed and approved by the Service de la consommation et des affaires vétérinaires (SCAV – EXPANIM) of the Canton of Vaud, Switzerland.

## Author Contributions

HY, AC, and JS contributed to the conception and design of the study. HY performed the experiments, analyzed the data, and drafted the manuscript. All authors contributed to manuscript revision and approved the submitted version.

## Conflict of Interest

At the time of manuscript preparation and submission, AC was employed by General Electric Medical Systems, Inc., which has commercial interests in the clinical application of hyperpolarized ^13^C metabolic imaging technology. The remaining authors declare that the research was conducted in the absence of any commercial or financial relationships that could be construed as a potential conflict of interest.

## Publisher’s Note

All claims expressed in this article are solely those of the authors and do not necessarily represent those of their affiliated organizations, or those of the publisher, the editors and the reviewers. Any product that may be evaluated in this article, or claim that may be made by its manufacturer, is not guaranteed or endorsed by the publisher.

## Footnotes

1
https://bayesiananalysis.wustl.edu

2
https://www.r-project.org

## Abbreviations

PDH: pyruvate dehydrogenase
PEPCK: phosphoenolpyruvate carboxykinase
PC: pyruvate carboxylase
AST: aspartate aminotransferase
3-MPA: 3-mercaptopicolinic acid.
