# Supplementary material for: Assessment of Aspartate and Bicarbonate Produced From Hyperpolarized [1-13C]Pyruvate as Markers of Renal Gluconeogenesis
Source: Front Physiol. 2021 Dec 10;12:792769. doi: 10.3389/fphys.2021.792769 (PMC8702956; doi:10.3389/fphys.2021.792769)
Supplement: Supplementary file 1 [file Data_Sheet_1.PDF]

Supplementary Material

Assessment of aspartate and bicarbonate produced from hyperpolarized [1-^{13}C]pyruvate as markers of renal gluconeogenesis

Hikari A. I. Yoshihara[1*], Arnaud Comment[2,3], Juerg Schwitter[4,5]

[1]Laboratory for Functional and Metabolic Imaging, Institute of Physics, EPFL (Swiss Federal Institute of Technology), Lausanne, Switzerland,

[2]Cancer Research UK Cambridge Institute, University of Cambridge, Cambridge, UK

[3]General Electric Healthcare, Chalfont St Giles, UK

[4]Division of Cardiology, Lausanne University Hospital (CHUV), Lausanne, Switzerland

[5]Cardiac MR Center, Lausanne University Hospital (CHUV) and University of Lausanne (UNIL), Lausanne, Switzerland

1 Supplementary figures

Figure S1

Figure S2

Figure S3

Figure S4

1.1 Supplementary Figures

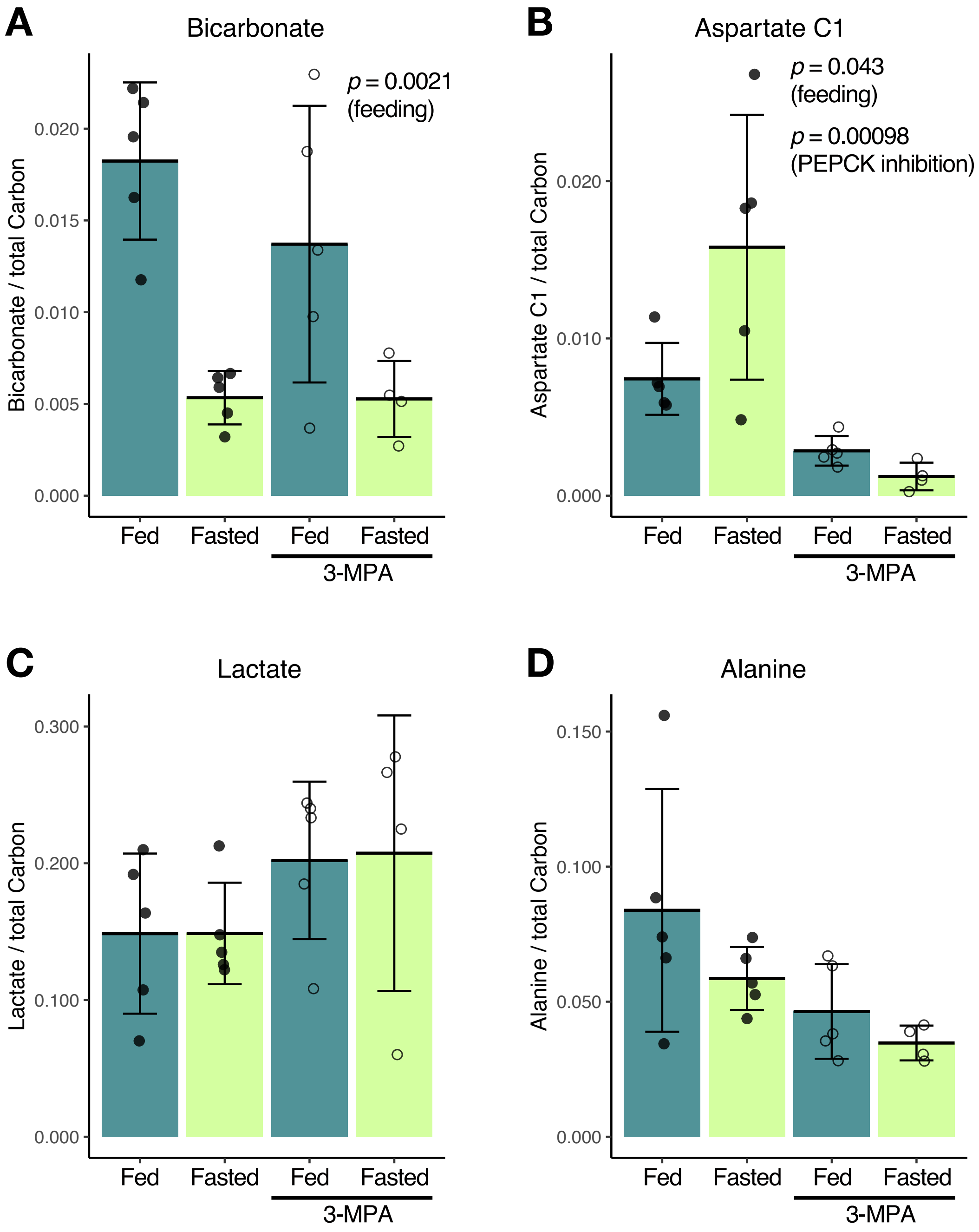

Figure S1. Effect of fasting and PEPCK inhibition by 3-MPA on renal conversion of [1-^{13}C]pyruvate to bicarbonate (A), aspartate (B), lactate (C), and alanine (D). Metabolite-to-total-carbon signal ratios (including pyruvate) are calculated from the fitted spectral amplitudes of summed spectra from each infusion series. Filled dots represent values from untreated rats, while open circles are from 3-MPA-treated rats. The indicated *p* values were calculated by two-way ANOVA, and they note the factor responsible for the significant difference.

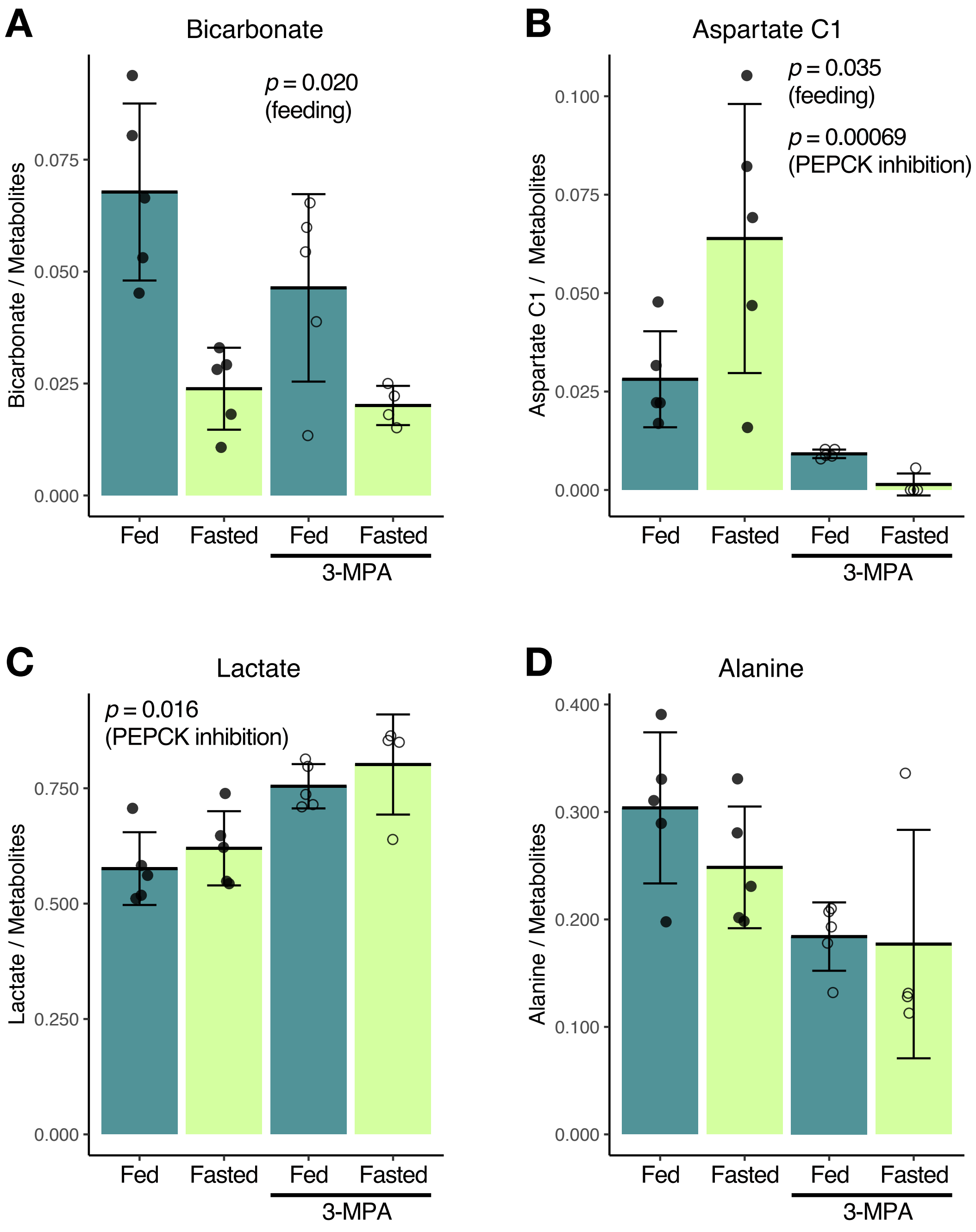

Figure S2. Effect of fasting and PEPCK inhibition by 3-MPA on renal conversion of [1-^{13}C]pyruvate to bicarbonate (A), aspartate (B), lactate (C), and alanine (D). Metabolite-to-total-metabolite signal ratios are calculated from the area under the curve (AUC) of fitted spectral amplitudes of individual spectra from each infusion series and the time of acquisition. Filled dots represent values from untreated rats, while open circles are from 3-MPA-treated rats. The indicated *p* values were calculated by two-way ANOVA, and they note the factor responsible for the significant difference.

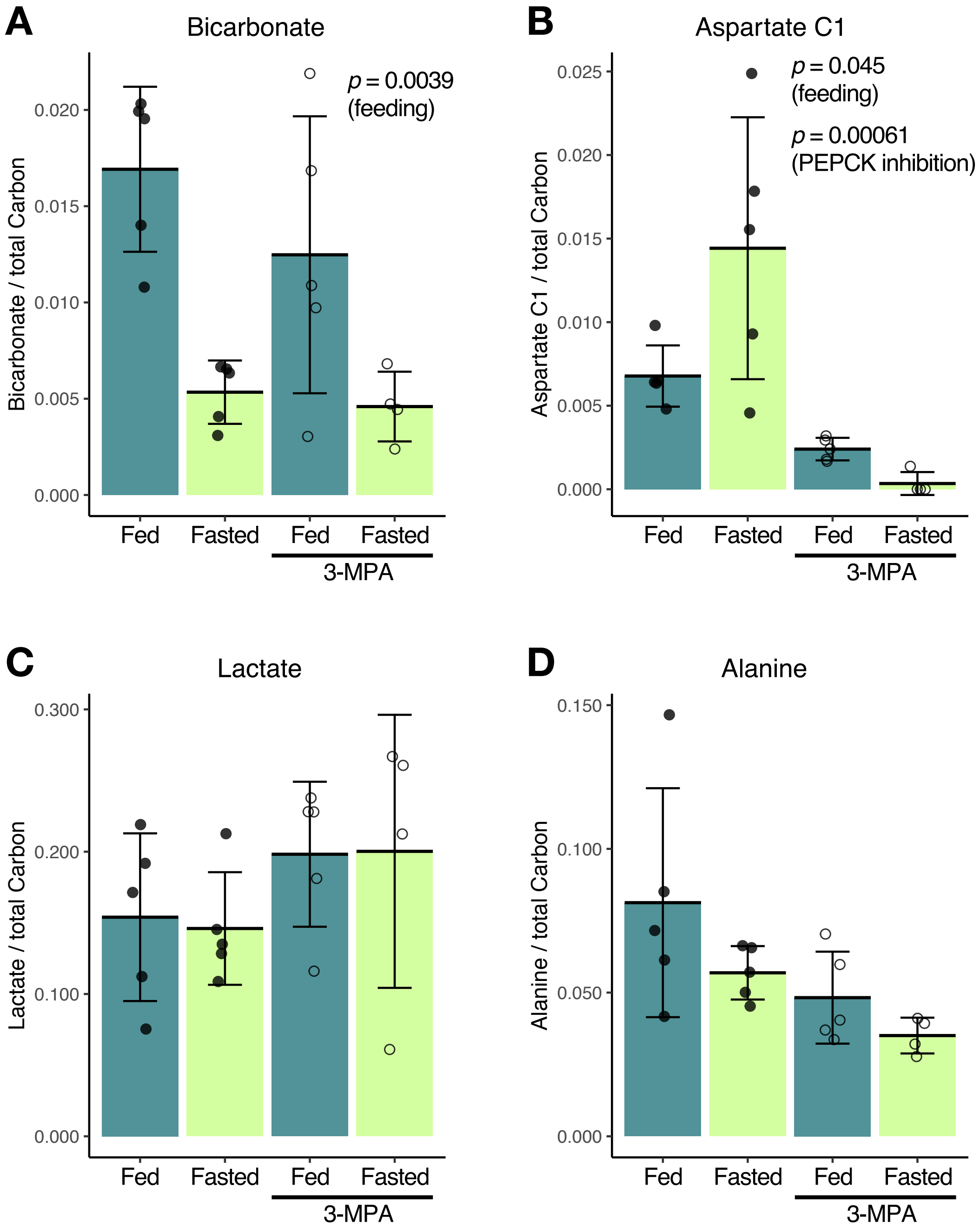

Figure S3. Effect of fasting and PEPCK inhibition by 3-MPA on renal conversion of [1-^{13}C]pyruvate to bicarbonate (A), aspartate (B), lactate (C), and alanine (D). Metabolite-to-total- carbon signal ratios (including pyruvate) are calculated from the area under the curve (AUC) of fitted spectral amplitudes of individual spectra from each infusion series and the time of acquisition. Filled dots represent values from untreated rats, while open circles are from 3-MPA-treated rats. The indicated *p* values were calculated by two-way ANOVA, and they note the factor responsible for the significant difference.

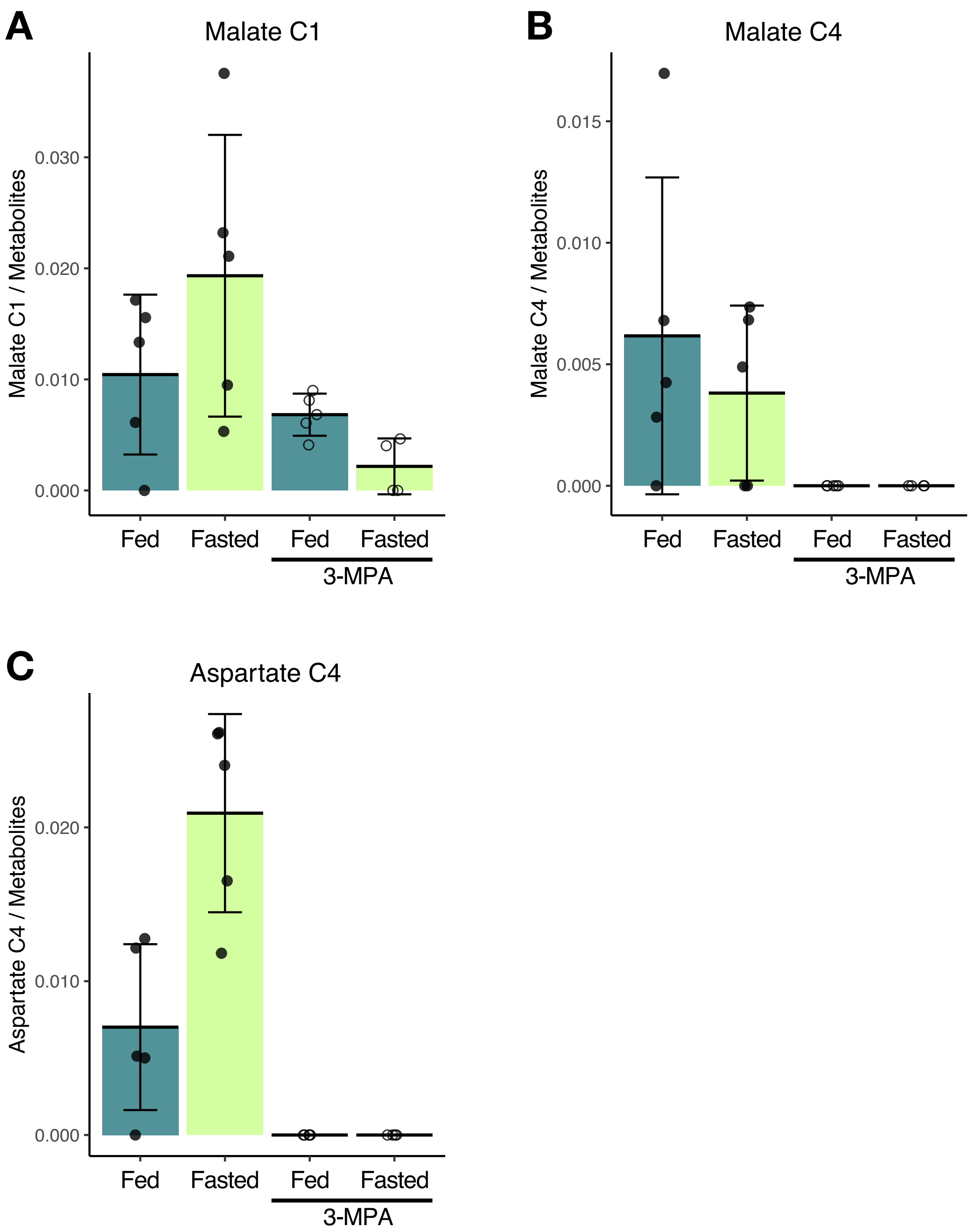

Figure S4. Effect of fasting and PEPCK inhibition by 3-MPA on renal conversion of [1-^{13}C]pyruvate to malate C1 (A), malate C4 (B), and aspartate C4 (C) Metabolite-to-total-metabolite signal ratios are calculated from the fitted spectral amplitudes of summed spectra from each infusion series. Filled dots represent values from untreated rats, while open circles are from 3-MPA-treated rats. Points with nominally zero signal indicate either a low signal for which a reliable fit could not be achieved or the absence of a visible spectral peak.
